# A Robust Real-Time Detecting and Tracking Framework for Multiple Kinds of Unmarked Object

**DOI:** 10.3390/s20010002

**Published:** 2019-12-18

**Authors:** Xiaodong Lv, Chuankai Dai, Luyao Chen, Yiran Lang, Rongyu Tang, Qiang Huang, Jiping He

**Affiliations:** 1 Beijing Advanced Innovation Center for Intelligent Robot and System, Beijing Institute of Technology, Beijing 100081, China; xiaodong.lv@bit.edu.cn (X.L.); 3120160125@bit.edu.cn (C.D.); qhuang@bit.edu.cn (Q.H.); jiping.he@bit.edu.cn (J.H.); 2 School of Optical and Electronic Information, Huazhong University of Science and Technology, Wuhan 430074, China; chenluyao1991@hust.edu.cn

**Keywords:** Rat-YOLO, Kalman filter, nine-point position correction, automatic generating label datasets, highlight removal

## Abstract

A rodent real-time tracking framework is proposed to automatically detect and track multi-objects in real time and output the coordinates of each object, which combines deep learning (YOLO v3: You Only Look Once, v3), the Kalman Filter, improved Hungarian algorithm, and the nine-point position correction algorithm. A model of a Rat-YOLO is trained in our experiment. The Kalman Filter model is established in an acceleration model to predict the position of the rat in the next frame. The predicted data is used to fill the losing position of rats if the Rat-YOLO doesn’t work in the current frame, and to associate the ID between the last frame and current frame. The Hungarian assigned algorithm is used to show the relationship between the objects of the last frame and the objects of the current frame and match the ID of the objects. The nine-point position correction algorithm is presented to adjust the correctness of the Rat-YOLO result and the predicted results. As the training of deep learning needs more datasets than our experiment, and it is time-consuming to process manual marking, automatic software for generating labeled datasets is proposed under a fixed scene and the labeled datasets are manually verified in term of their correctness. Besides this, in an off-line experiment, a mask is presented to remove the highlight. In this experiment, we select the 500 frames of the data as the training datasets and label these images with the automatic label generating software. A video (of 2892 frames) is tested by the trained Rat model and the accuracy of detecting all the three rats is around 72.545%, however, the Rat-YOLO combining the Kalman Filter and nine-point position correction arithmetic improved the accuracy to 95.194%.

## 1. Introduction

In order to study the relationship between neural activity and behavioral activity, video image processing is necessary [1,2]. The social interaction (SI) is an important means to study anxiety in rats [3], and the behavioral characteristics of anxiety can be identified by some behavioral actions, such as sniffing partners, climbing over, crawling under, mutual grooming, genital investigation and following, and walking around. Autism, poor communication among young children, and poor social skills are the commonly studied questions, in this area as these factors jeopardize the development of the behavior and psychology of children, meaning the children cannot express their mental activities effectively. Therefore, the early detection of autism and timely intervention can reduce the damage to children with autism [4]. As the social behavior is more complex than we can image, some works focus on the interaction between two rats [5,6], and real-time target tracking throughout the process provides a more comprehensive analysis of animal neural activity in studying animal neural activity. In the past survey of social behavior, the focus of research has been the interaction between the two rats in a cage [7,8]. Therefore, the research of social behavior with more than two rats is a good research direction to study SI in the future.

The development of machine vision has been ongoing rapidly along with the development of deep learning in recent years. The current detection method of rats is based on the traditional image processing methods, such as background subtraction [9,10], but by this method, the background is generally set as simple, and there is a great contrast between the detected object and background in the histogram of the image. However, some social behavior requires experiments to be in different scenes, and the experimental background is dynamic rather than static; thus, the method make it more possible to recognize fault objects in the condition, Furthermore, using more complex arithmetic solves this problem which may consume more time. In conclusion, it is necessary to develop robust object detecting and tracking software.

Deep learning in machine vision is a better method than others in multi-object detection (MOD); in particular, the convolutional neural network (CNN) is used in image recognition. Alex proposed AlexNet [11], which is designed by CNN and won the championship in the ImageNet LSVRC-2010 contest including 1.2 million high-resolution models and 1000 different classes. In AlexNet, rectified linear units (ReLUs) are used for the activation function of neurons to shorten the calculation time, and Dropout [12] is adopted in the framework to avoid overfitting. Next, in the ImageNet Large-Scale Visual Recognition Challenge 2014 (ILSVRC14), VGGNet secured the second places with a lower error rate than AlexNet, which increased the depth using an architecture with very small (3 × 3) convolution filter [13]; moreover, GoogLeNet [14] won the championship with a 6.67% error rate, replacing three full connection layer with an average pooling layer to improve accuracy in the deep learning framework, and in order to avoid the disappearance of gradient, during the training, two auxiliary linear layers with softmax loss as the classifiers were used to output one of the middle layers as a classification with a little weight (the losses of the auxiliary classifiers were weighted by 0.3). Next, the mask R-CNN [15] is a classic multi-target recognition framework; however, it cannot process images in real time, and the SSD (single shot multi-box detector) [16] with the single shot detection model has a relatively faster recognition speed. However, Joseph Redmon proposed a YOLO v3 framework [17] in 2018 which is better than YOLO v2 [18] at small object detection, faster than Mask-RCNN, and has higher detection accuracy than DSSD (Deconvolutional Single Shot Detector) [19]. The deep learning framework has been used in some studies in different fields, for example, Koirala [20] used the framework to estimate the yield of mango, Zhang [21] used the framework to detect the lane in real time, and Tian [22] used the framework to detect apples in real time during different growth stages. In this study, we apply the YOLO v3 in our project as the state-of-art detection and localization algorithm for rats.

As it is inevitable that it is impossible to detect objects in every frame, for the position prediction in the next frame, it is necessary to fill the object in the image. With lower memory usage and faster computing speed, the Kalman Filter [23] provides the means of target state prediction in a continuously changing system, and when some person box measurements are not available (due to occlusion or merge problems), Girondel proposes a method of real-time tracking for multiple persons and their faces simultaneously in a video sequence using the Kalman Filter [24]. Bewley proposed a simple online and real-time tracking model to track objects using the Kalman Filter and Hungarian algorithm [25] and the tracker updates at a rate of 260 Hz, which is more than 20 times faster than other state-of-the-art trackers [24]. Therefore, the Kalman Filter is a better way to fill the objects lost in the current frame, and the nine-point position correction algorithm is proposed to verify the correctness of the predictable location and fine-tune the center to a suitable location based on grayscale.

As deep learning needs more datasets to train the model, the datasets need to be marked by human, which is a time-consuming work, and finding a full-time employee to mark the data set requires a large amount of money. Besides this, for a specific object detection model, marking and data training are essential tasks, and software needs to be essential to designed to generate a labeled dataset. In this study, new software for automatic marking is designed, which provides an accurately labeled dataset for YOLO training. The bilateral filter [26] is applied in the software to remove “salt and pepper”, which can smooth images while preserving edges. In order to enhance the contrast of the image, the histogram equalization is a better way to solve the problem and the adaptive histogram equalization [27] has advantages in avoiding the over-enhancement of noise. Edge detection is a better object detection method in automatic marking, such as with a canny edge detector or Sobel edge detector. Besides this, the automatic marking software proposed in this project can be corrected manually by labelImg (https://github.com-/tzutalin/labelImg).

In the field of machine vision, the highlight of a textured object is a linear combination of specular reflection and diffuse reflection. Specular reflection is the most destructive factor of object detection can cause image brightness saturation and detection error. Thus, it is useful, meaningful, and greatly important to remove the specular reflection with a fast speed and high quality. In some previous works, using many images of the same scene is a common method to remove specular reflection; however, this method requires too many of images of the same scene and it is difficult to perform [28,29,30]. In 2005, Tan [31] proposed the concept of a specular-free image and removed highlights using a single image; however, the disadvantage is that the method is time-consuming. Next, Yang [32] added bilateral filtering into real-time specular highlight removal, running over 200× faster than the state-of-the-art algorithm of the time, but in our study, as this method removes highlights by iterations and consumes a large amount of time, the local illumination changes [33] apply an non-linear transformation to the gradient field inside the selection and then integrate back with a Poisson solver, locally modifying the apparent illumination of an image. Further, multi-threading is added to local illumination changes over a short time. 

In summary, the main advantages of the study are as follows:

1. The structure of the real-time detector-tracker of a rat is composed of rat-YOLO, the Kalman filter, Hungarian algorithm, and nine-point fine position correction to identify, predict, and track rats in a fixed scene. Besides this, it achieves offline object tracking.
2. Nine-point fine position correction is proposed in this study to correct the target position. As the predicted target position of the Kalman Filter is not necessarily accurate, the correction algorithm is proposed to verify the correctness.
3. An automatic marking software of rat label images is proposed. The software is limited in generating rat labels under a simple scene, and the labeled dataset can be used in the YOLO model training.
4. A multithreading local removal highlighting algorithm to remove highlights is proposed in this paper, which can remove highlights in a fixed region and save time.

The structure of the paper is as follows. In Section 2, we introduce the building of the experimental environment, which includes the experimental animals, camera hardware and detection algorithm. In Section 3, we present the methods for building the detecting and tracking framework, and the software of auto-generation labeled datasets. In Section 4, we introduce the results and discussions of the rat detection. In Section 5, we present the conclusions of the detecting and tracking framework.

## 2. Materials

### 2.1. Animals Selection

Three healthy 8-week-old male Sprague Dawley rats (weighting 330–350 g) were selected in the experiment. The rats were housed at 30 °C, with 55.3% humidity and a 12-h light/dark cycle, with access to food and water ad libitum. Food and the water were fully available in the cage (product number: HH-MMB-1). All animal experiments were performed in accordance with the Guide for the Care and Use of Laboratory Animals [34]. The procedures in the study were designed to minimize the pain or discomfort of the animals, in accordance with the current protocols approved by the Laboratory Animal Ethics Committee of Beijing Institute of Technology (Beijing, China).

### 2.2. Hardware Platform

**Figure 1:**
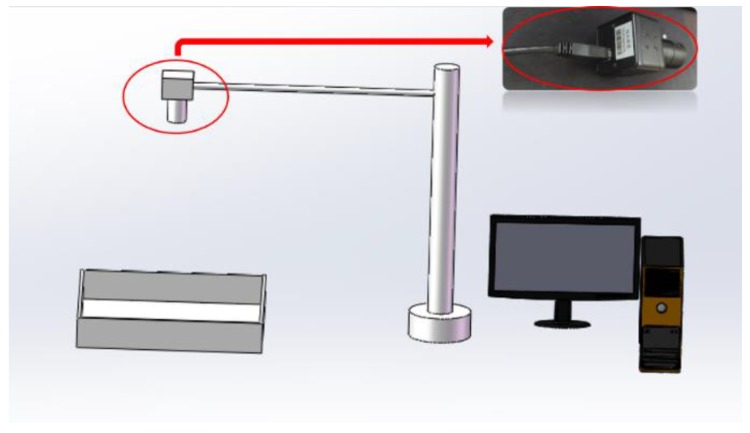
The schematic diagram of the experimental device which includes a mine, an industrial CMOS camera, and a data processing computer. The size of the mine is 100 mm × 85 mm and the height of the camera is 180 mm.

The video was captured by an industrial CMOS (Complementary Metal-Oxide-Semiconductor, CMOS) camera (maximum 30 fps, operated at 10 fps, 1024 × 760 pixels), The camera was set at a F/# of 1.8 with an exposure of 2.5 ms for the capture of video. The size of the mine was 100 mm × 85 mm and the height of the camera was 180 mm. The Rat-YOLO deep neural network was trained and run on the same platform (HP Pavilion Gaming Desktop 790-08xx, Palo Alto, CA, USA) with the Intel^®^ CoreTM i7-8700 CPU@3.20 GHz, 16 GB RAM, NVIDIA GeForce RTX 2070 GPU (1620 MHz GPU clock) with 8 GB dedicated memory, 64 bit Windows 10 Family Chinese Edition, CUDA v9.0.176, cuDNN v7.0.5, Python 3.5.6 and Opencv v4.1.0.25. The video capture platform is shown in Figure 1 and the activity of the rat was captured in a bird-view image.

## 3. Methods

### 3.1. Rat YOLO Detector and Tracking

**Figure 2:**
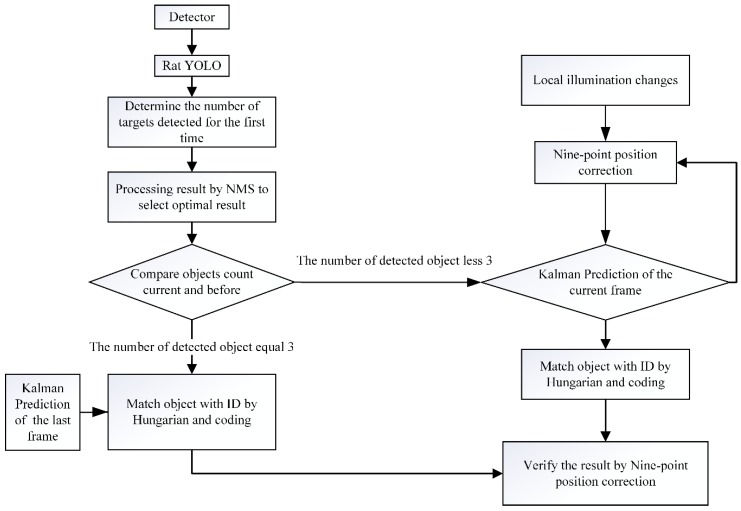
Flow diagram of the real-time objects detecting and tracking software. YOLO: you only look once.

Firstly, the detection and prediction modules and the detection flow are illustrated in Figure 2. The Rat-YOLO detection aims to detect the objects, return the number of detected objects and the location of each objects in the video frame. The YOLO v3 [17] was trained on the Microsoft COCO dataset [35] which contains 80 objects except the rats and has a good performance in terms of detection speed and accuracy. In our project, the model was trained based on the trained weighted results. The Rat-YOLO model proposed in the study is trained by the marked images which are generated by the automatic label system and generated by manual correction. 

Secondly, in the local object detection, the same target may correspond to multiple bonding boxes which are detected by Rat-YOLO. In order to keep the box with the highest score and the most appropriate location, non-maximum suppression [36] is adopted as a method to optimize the candidate box. According to the score, the extra boxes with a large overlap area will be deleted.

The step of non-maximum suppression is as below:

(1) Calculate the area of every bonding box and sort by score.
(2) Calculate the intersection over union (IOU), for which the equation is shown in Equation (1).
(3) If the value of IOU exceeds the threshold, the bonding box with a low score is deleted.

(1) $$\mathrm{IOU}=\frac{area\left(ROI_{T}\cap ROI_{G}\right)}{area\left(ROI_{T}\cup ROI_{G}\right)}$$

### 3.2. Nine-Point Position Correction Algorithm

**Figure 3:**
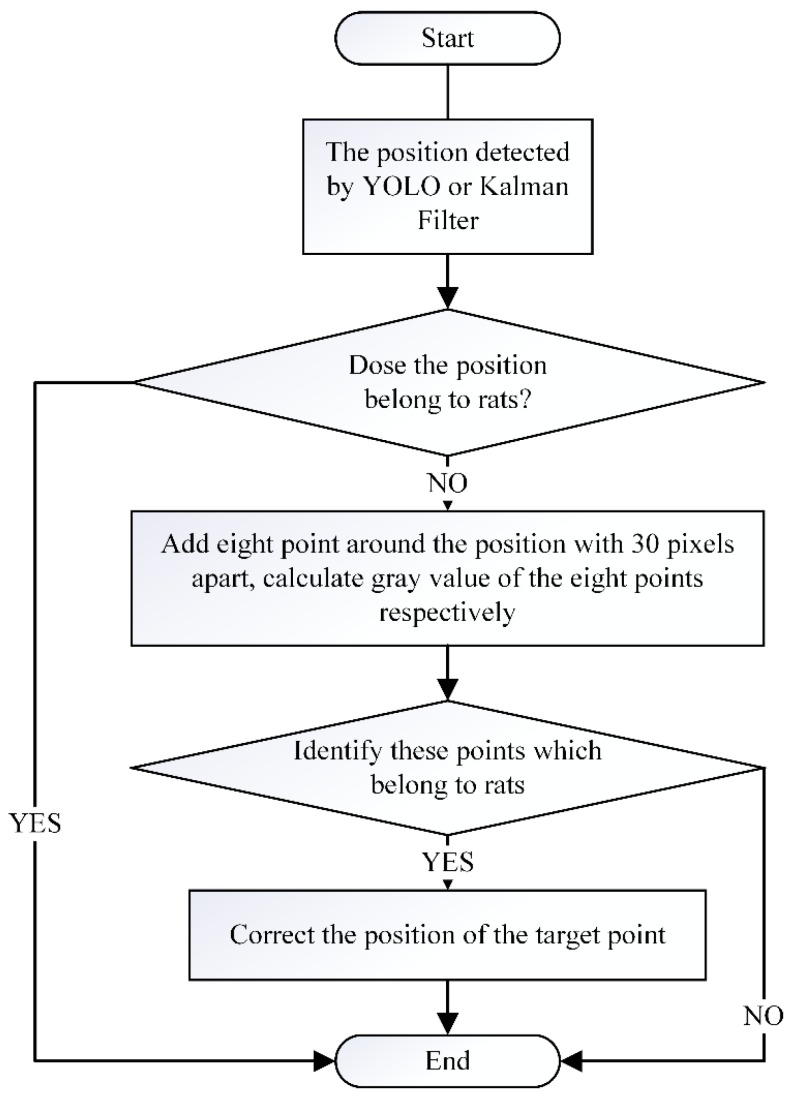
Flow diagram of the nine-point position correction algorithm.

**Figure 4:**
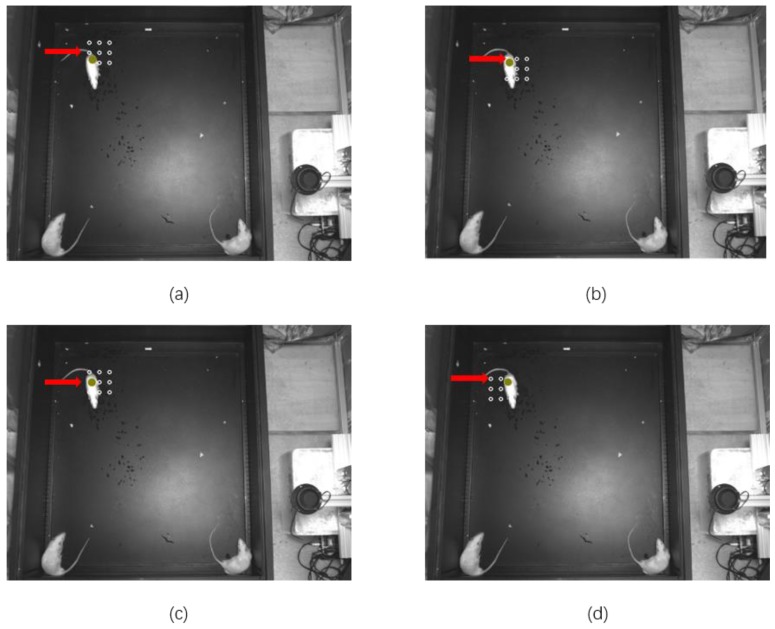
Partial correction situation of the nine-point position correction algorithm; white represents original points, yellow represents corrected tracking points; (**a**) the center position of the nine points is not on the body of rats and the left and bottom of the nine points is on the body of rats; (**b**) the center position of the nine-point is not on the body of rats, and the left and top of the nine-point is on the body of rats; (**c**) the center position of the nine points is not on the body of rats and the left and median of the nine points is on the body of rats; (**d**) the center position of the nine points is not on the body of rats and the right, and top of the Nine-point is on the body of rats.

Since there are some errors in the data processing and uncertain factors, the predicted target is not necessarily the real object; thus, the nine-point position correction algorithm is proposed for the fine-tuning of the location of tracking objects, and the method is necessary to add in the object predicted arithmetic to verify the predicted location. The flow diagram of the nine-point position correction algorithm is shown in Figure 3. As shown in Figure 4, eight points around the one point chosen from the location detected by Rat-YOLO or the location predicated by the Kalman Filter are added in the gray image, and the space of every pixel is 30. Then, we calculate the gray value of each point and compare the gray value with a fixed threshold (105) to identify whether the point belongs to the body of a rat. The mistaken points are modified in the yellow point in Figure 4. For example, as shown in Figure 4a, the location of the Rat-YOLO detector or Kalman Filter is not on the body of rats, therefore, in this situation, the gray value of all the nine points are calculated according the comparison of every gray value of each point with each other and the experiential gray value of the rat’s body. Then, an optimal point is selected from the nine points, and the result is shown in Figure 4a. The location of the optimal point is represented by the yellow circle.

### 3.3. Rat Kalman-Filter-Model

A necessary problem is the inference of the motion of one or more objects from a sequence of images and it is inevitable that detection error appears in the detection processing of rat YOLO. Therefore, a Rat Kalman-Filter-model [23,25] is added to predict the missed rat location in the next subsequent frame. The application of the Kalman Filter in the project is used to provide the location of objects, which is used to fill the losing location of objects and provide evidence to associate the ID number of objects between different frames. The Kalman Filter runs in a two-step recursive process. The first step is to predict a new state with the uncertainties, and the second step is to update the current optimal value according to the measurement of the current frame and the new state obtained from the first step. In the project, we assume that the rat motion model is a linear constant velocity model:

(2) $$\left[\begin{array}{c} \begin{array}{c} x_{t+1}\\ y_{t+1} \end{array}\\ \begin{array}{c} v_{x,t+1}\\ v_{y,t+1} \end{array} \end{array}\right]=\left[\begin{array}{cc} \begin{array}{cc} 1 & 0\\ 0 & 1 \end{array} & \begin{array}{cc} dt & 0\\ 0 & dt \end{array}\\ \begin{array}{cc} 0 & 0\\ 0 & 0 \end{array} & \begin{array}{cc} 1 & \;0\\ 0 & \;1 \end{array} \end{array}\right]\left[\begin{array}{c} \begin{array}{c} x_{t}\\ y_{t} \end{array}\\ \begin{array}{c} v_{x,t}\\ v_{y,t} \end{array} \end{array}\right]+w\left(t\right)$$

where $x_{t+1}$ and $y_{t+1}$ represent the object’s horizontal and vertical location in the next frame respectively, $v_{x,t+1}$ and $v_{y,t+1}$ represent the horizontal and vertical velocity, $x_{t}$ and $y_{t}$ represent the object’s horizontal and vertical location in the current frame, $v_{x,t}$ and $v_{y,t}$ represent the horizontal and vertical velocity, respectively, and w(t) represents the noise covariance matrices.

### 3.4. Improved Hungarian Filter Model

**Figure 5:**
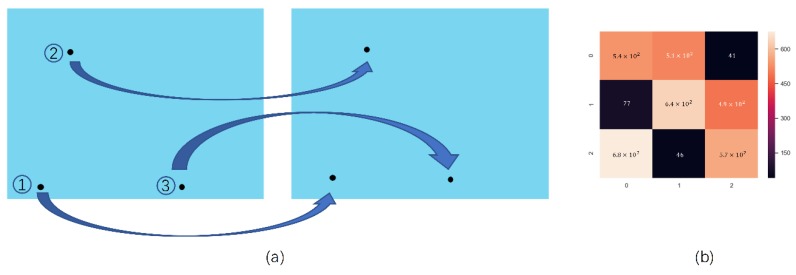
A common situation of Hungarian Filter’s application; (**a**) the object location of the current frame and next frame; (**b**) a matched form between the current frame with next frame calculated by the Hungarian Filter, the label on the left represents the ID number of current frame, the label on the bottom represents the ID number of current frame, and the score represents the suitability between the last frame with the next frame.

**Figure 6:**
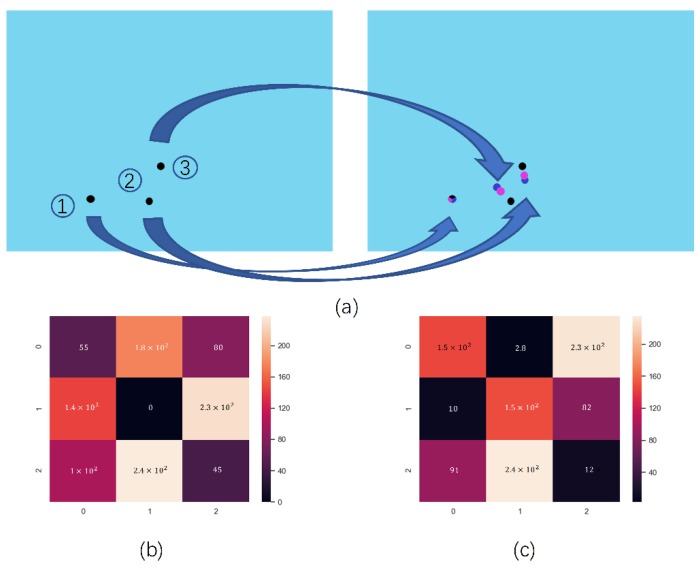
Adding the location of Kalman Filter and Rat-YOLO to calculate the form of the Hungarian Filter; (**a**) red represents the location of detected by Rat-YOLO, blue represents the location of pridicted by the Kalman Filter, and black represents the object location of the last frame; (**b**) a matched form between the current frame with Rat YOLO’s result of the next frame calculated by the Hungarian Filter; (**c**) a matched form between the current frame with Kalman Filter’s result for the next frame calculated by the Hungarian Filter.

After the objects have been detected by the detector and Kalman Filter, the problem of assigning the object of the current frame with object of next frame need to be solved. The aim of finding the relation of detected objects between two frames is to find the combination of minimum Euclidean distance. Thus, the Hungarian filter model is used in the project to match the number of each frame. In the study, there are three objects in every frame, and the number is made up in the first frame. Then, the next frame of objects is numbered according to the minimum sum of the Euclidean distance and the number of the previous frame’s objects. As shown in Figure 5a, it is a common scene to match the object of next frame with the current frame, and in this scene, the coordinate of the detected object in the current frame and the next frame is used to calculate the minimum Euclidean distance. The score of the matched form calculated by the Hungarian filter is shown in Figure 5b, and according to the minimum distance sum, the ID number of the next frame is matched to the ID number of the current frame. However, as shown in Figure 6a, this a special scene. In this scene, one of the objects has three labeled moves to the place close to another object with two labeled moves, and the object has two labeled moves to the place close to another object with three labeled moves in the next frame, meaning that the traditional Hungarian Filter will calculate the wrong result, which is shown in Figure 6b. So, in this scene, the result of the Kalman Filter is added to calculate the score of the Hungarian Filter, and the corrected score is shown in Figure 6c.

### 3.5. The Multithreading Local Removal Highlighting Algorithm

**Figure 7:**
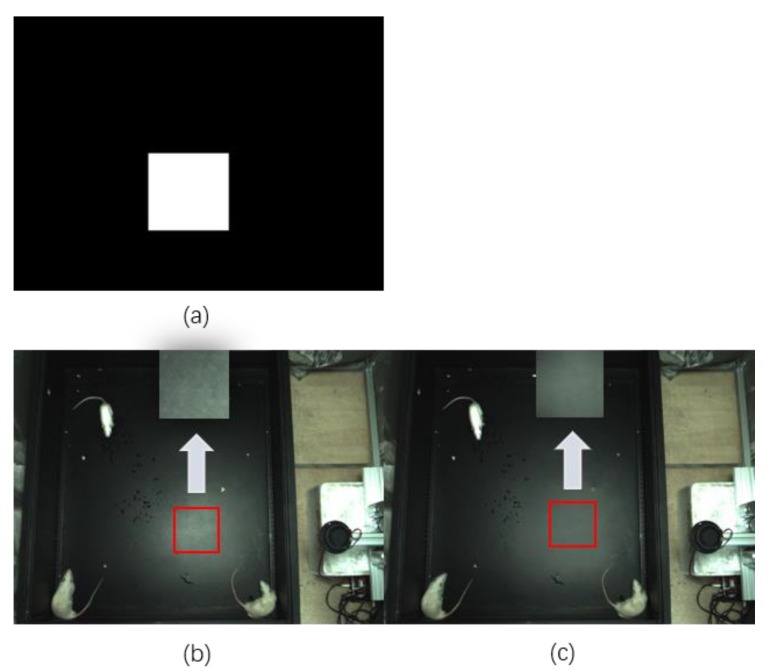
Local illumination changes to remove highlights; (**a**) removal highlight mask; (**b**) original highlight area; (**c**) the highlight area processed by the local illumination changes.

Light illuminates the surface of the object to produce specular and diffuse reflections and the specular reflection is a very serious obstacle in the target detection task [37,38]. As shown in Figure 7b, there is a big highlight area in the middle of the image; thus, a method to reduce specular reflection in real time is necessary. As the experiment scene is fixed in our study and the intensity of the light is constant, the area size of specular reflection is relatively constant. Firstly, an image was pretreated, and a region of specular reflection was taken to reduce the area of processing, which is shown in Figure 7a. In order to obtain the advantage of removing specular reflection at speed, a multithreading local illumination changes [33] is adopted in the project. The process is processed in parallel with Rat-YOLO; thus, it saves the time. Based on the test and experiment, the α and the β are both 0.4 and the algorithm is given in the following (Algorithm 1).

**Algorithm 1** The Multithreading Local Removal Highlighting Algorithmbegin:1. read original image, mask image, and channel = 0;   for (channel++ < 3):    2. the gradient field (v) of the logarithm of the image is transformed$v=\;\alpha^{\beta }{\left|\nabla f^{*}\right|}^{-\beta }\nabla f^{*\;}with\;\alpha =0.4,\beta =0.4$    3. Solve Δf=divv to recon- struct the logarithm of the image, f;  endend

### 3.6. Automatic Generating Labeled Dataset

The training of the CNN model requires a great number of marked datasets; there are no open source datasets, especially in a specific project, and there are some disadvantages in manual marking, such as the consumption of time and money. To the authors’ knowledge, there is no software to automatically generate labeled datasets of rats in the PASCAL VOC format. In the current study, the marked datasets of Sprague Dawley (SD) rats are rare and not opensource, and Zhang proposed a method for the automatic generation of the lane label images [21]. Thus, a method to automatically generate marked datasets for SD rats in a fixed simple scene was proposed and in order to avoid some mistakes in the automatic system and increase the accuracy of marking, the generated datasets can be manually modified using the LabelImg software.

An automatic marking system is proposed in the paper, and the algorithm of the detection of rats in shown in Algorithm 2. To remove “salt and pepper” and smooth the image, the Gaussian filter is applied in the study. The σ is calculated by

(3) σ=0.3∗((ksize−1)∗0.5−1)+0.8,

where ksize in the equation is set to 5. If we turn up the σ, the effect of distant pixels on the center pixel increases. According to practical testing in our study, the σ is set to 1.1. The kernel size of the filter is 5 × 5, which is used to smooth the image. A one-dimensional Gaussian function is described in the Equation:

(4) $$G_{\left(x\right)}=\frac{1}{\sqrt{2\pi }\sigma }e^{-\left(x^{2}\right)/2^{2}}$$

where σ is calculated by Equation (1). Then, a two-dimensional Gaussian function is described in the Equation

(5) $$K_{\left(x,y\right)}=G_{\left(x\right)}*(G_{\left(x\right)}.T)$$

and the Gaussian Kernel is calculated from the equation. A new pixel $Q_{\left(x,y\right)}$ is calculated by the Equation

(6) $$Q_{\left(x,y\right)}=\overset{2}{\underset{j=-2}{\sum }}\overset{2}{\underset{i=-2}{\sum }}P_{\left(x+i,y+i\right)}*K_{\left(x+i,y+i\right)}$$

where, in this project, the $P_{\left(x+i,y+i\right)}$ Table 1 and the Table 2 are shown below:

In order to avoid excessive filtering by the Gaussian filter, the bilateral filters [26] are applied in the second filtering to remove noise, smooth small fluctuation in the image, and preserve edges. In the bilateral filter, the weighting of grayscale information is added to the Gaussian filter. In the neighborhood, the point close to the gray of the center point has a higher weight; in contrast, the weight is smaller. The gray distance is calculated by the Equation:

(7) $$grayDistance_{\left(x,y\right)}=\frac{1}{2\pi \sigma_{1}^{2}}e^{-{\left(gray\left(x_{i},y_{i}\right)-gray\left(x_{c},y_{c}\right)\right)}^{2}/2\sigma_{1}^{2}}$$

The spatial distance is calculated by the Equation:

(8) $$spaceDistance_{\left(x,y\right)}=\frac{1}{2\pi \sigma_{2}^{2}}e^{-({\left(x_{i}-x_{c}\right)}^{2}+{\left(y_{i}-y_{c}\right)}^{2})/2\sigma_{2}^{2}}$$

**Figure 8:**
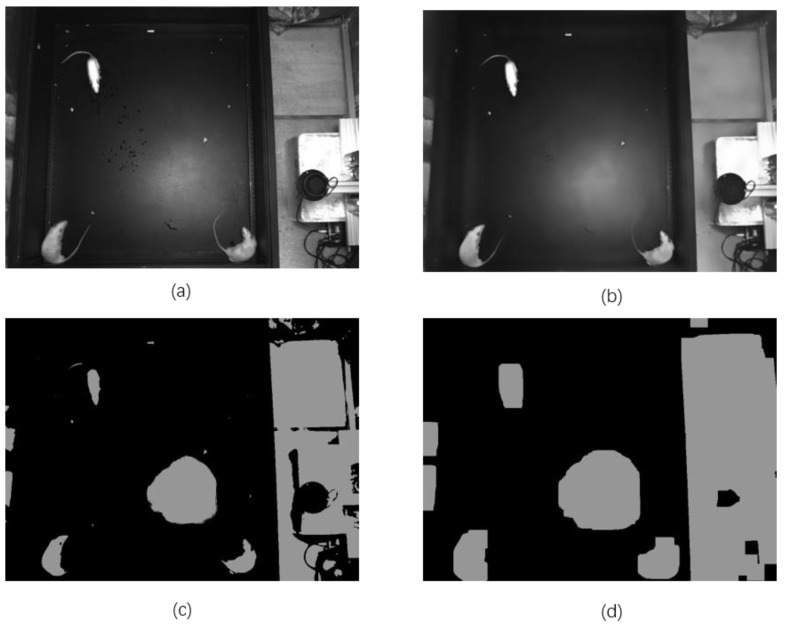
The original image and results with different image filter (**a**) original image; (**b**) image after Gaussian filter and bilateral filters; (**c**) image after Binarization with the parameter (100); (**d**) image after erosion and dilation, both using a 5 × 5 rectangular structuring element.

According to the practical testing in our study, the $\delta_{1}$ of color is set to 75, the $\delta_{2}$ of space is set to 75, and the diameter around each pixel area is set to 40 when filtering. As shown in Figure 8a,b, after Gaussian filtering and bilateral filtering, the image becomes smooth and shows less white noise, which reduces the difficulty of the object edge recognition.

**Algorithm 2** The Flow of Generating Labeled Datasetbegin:1. Collect a 500-frame video under a fixed scene;   while (frame.num++ <= frame.total_num):
   2.Read video by frame;   3.Gaussian filter with the ksize of 5;   4.Bilateral filters with $\delta_{1}$ and $\delta_{2}$ both 75 and Diameter of each pixel neighborhood is set to 40;   5.Change color image to grayscale image;   6.Local Adaptive histogram equalization is applied to grayscale, Threshold for contrast limiting is set to 1; and the title grid size is set to 50 × 50;   7.Get a binary image out of a grayscale image and the threshold is set to 100;   8.Three iterations of erosion, followed by four iterations of dilations;   9.Limit maximum diameter of each object;   10.Output “.xml” files;
  end  11. open “.xml” files with LabelImg to modified the wrong datasets by human;end

Then, the image is converted to a gray image, and the adaptive histogram equalization is applied to the gray to improve contrast, which has the advantage of avoiding the over-enhancement of noise it produces in relatively homogeneous regions [27]. In the study, according to our experience, the value of clipped ahe (Adaptive Histogram Equalization) is set to 1.0 and the title grid size is set to 50 × 50. Next, the gray is processed by binarization using a fixed value of 100, and the result is shown in Figure 8c. However, there are some small noise areas in Figure 8c, which can influence the result of edge recognition. So, it is necessary to add a method to reduce these noises, such as a size limitation method and erosion-dilation method. In the project, the erosion-dilation method is used to reduce small noise, processing the image with three iterations of erosion, followed by four iterations of dilations to contain the whole body of rats as much as possible, both using a 5 × 5 rectangular structuring element and the result is shown in Figure 8d. In the figure, only some large amounts of noise remain. In the end, the maximum diameter of each object is calculated in Figure 8d, and a size limitation is adopted to remove large amounts of noise, and the limited rectangular is set to 200 × 200 based on our experimental data of rat morphology.

Finally, edge detection needs to be done for every frame. In 1985, Suzuki proposed a topological structural analysis of digitized binary imaged by border following [39]. The method finds the outlines of the rat from binarized image, removes the other outlines which are out of the range of the mine, and according to the morphological size of the rats, removes other interference factors. 

## 4. Results and Discussions

### 4.1. The Results of Automatically Generating a Labeled Dataset

**Figure 9:**
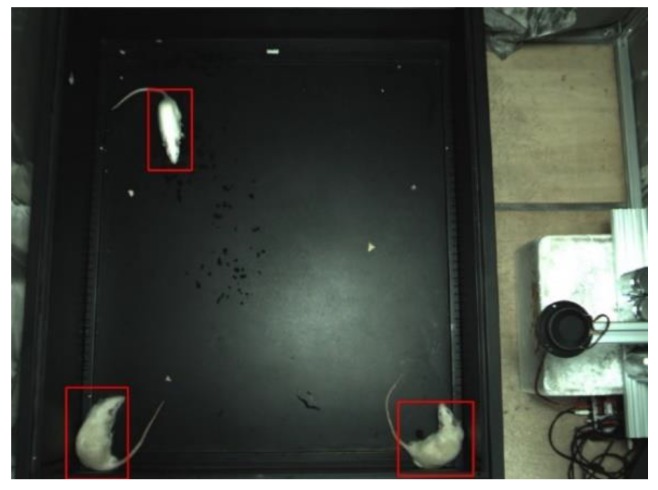
Automatically generated datasets shown in LabelImg.

To the authors’ knowledge, there is none software to automatically generate labeled datasets of rats in the PASCAL VOC format. As shown in Figure 9, the data of the maximum outer rectangle need to be calculated from image, which are used to train the Rat YOLO, and the red line is the calculated maximum outer rectangle. The marking data were calculated by the largest external quadrilateral of the contour and the data are output in xml files which are used to train the Rat-YOLO. The automatic generating datasets can be opened by the common well-known graphical image annotation tool LabelImg, whose annotations are saved as XML files in the PASCAL VOC format. The datasets generated by the automatically labeled software can be opened directly by LabelImg and the incorrect datasets can be modified to correct datasets by a human. 

As shown in Table 3, using the trained Rat-YOLO model based on the datasets generated from the automatic dataset generating software, the accuracy of all the rats detected is 72.545%, and only 25 frames out of 2892 frames did not detect any rats. So, up to a point, the Rat-YOLO model satisfies the goal of a detector. Besides this, in order to generate more generalized datasets, some methods such as image rotation, horizontal mirror, color balance processing brightness transformation, and blur processing can be used in the labeled datasets [22].

### 4.2. The Result of Missing Objects Filled by the Kalman Filter and Hungarian Filter Model

**Figure 10:**
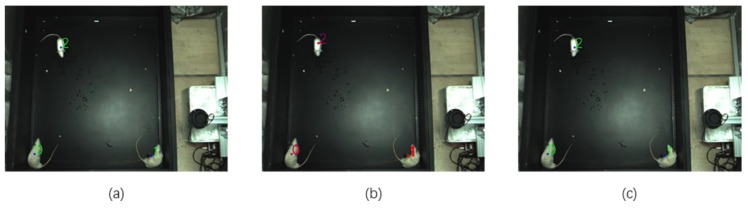
These is a sequence of images of rat’s movements; the green number indicates the right detector and tracking, and the red number indicates the corrected wrong data. (**a**) 37th frame, (**b**) 38th frame, (**c**) 39th frame.

The sequence images of rat movement from the 37th to 39th frames are shown in Figure 10. If the objects are detected by the Rat-YOLO detector, the color of the ID number labeled in the image is green. If the ID number is filled by the Kalman Filter, Hungarian Filter, and nine-point position correction algorithm, the color of the ID number labeled in the image is red. As we can see in Figure 10b, the detector didn’t detect all the objects in the 38th frame, but as shown in the Figure 10a,c, the framework detected all the objects in the previous frame and the next frame. As shown in Figure 10b, this framework can fill the losing ID number of rats.

### 4.3. The Accuarcy of Our Framework in Rat Tracking and Detecting

The best result for detector is detecting all the rats in every frame. However, in the experiment, it is impossible to detect all the rats in every frame. The frame is tested on a video with a total frame count of 2892. As shown in Table 3, no rats were detected is 0.864% of frames, the accuracy of one rat detected in the frame is 3.043%, the accuracy of one rat detected in the frame is 22.752%, the accuracy of all the rats detected in the frame is 72.545%, and the accuracy of more than three rats detected in the frame is 0.795%. The corrected data by the Kalman Filter, and nine-point position correction algorithm is output in an image of the “.jpg” format, and it can improve the accuracy to 95.194%, meaning the detecting accuracy has increased by 22.649%. The number of mistakes is counted by a human to calculate the error rate from the saved image, and in the error frames after correction, there are about 120 frames of data due to not detecting objects for a long time in the video.

### 4.4. Generate Exploration and Trajectory of the Rat

**Figure 11:**
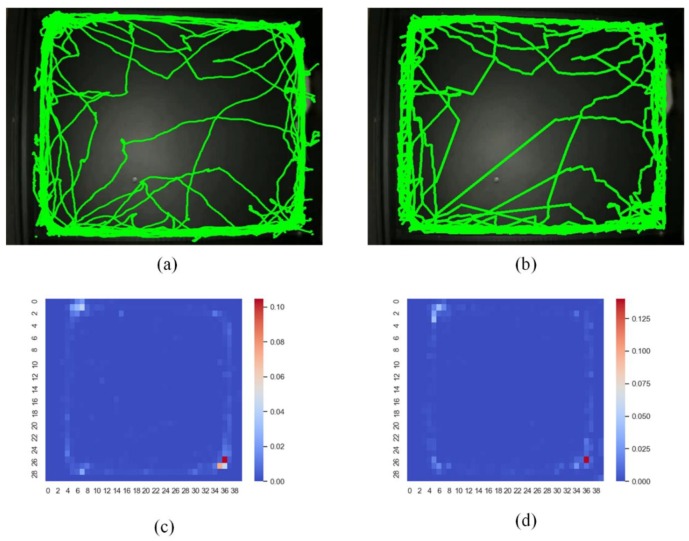
Trajectory and exploration of the rat in the mine—the color intensity represents the duration of stay; (**a**) trajectory drawn by Toxtrac; (**b**) trajectory drawn by ours methods; (**c**) exploration drawn by Toxtrac; (**d**) exploration drawn by ours methods.

The robust detecting and tracking system can plot the exploration of rats in the experiments. Recording the exploration of rats under a strange environment is an important method to study biophysiology, such as, infantile autism and anxiety disorder. In the comparison with the available tracking software Toxtrac [40,41], as shown in Figure 11, the trajectory and exploration of the rat in the mine can be improved. As shown in Figure 11a,b, the trajectory is plotted from a areal scene. The mine is divided into a 40 × 30 rectangular area and the statistics method is used to calculate the sum of frame of the rat appearing in the rectangular area. The video tracks the rat and records the trajectory, and they have the same amount of stay time, comparing Figure 11c with Figure 11d, and we can obtain the result that the rat likes to walk on the edge and the corner of the mine in a strange environment from the recorded trajectory.

## 5. Conclusions

As most behavior studies of rats concentrate on the interaction between two rats [5,6,7,8] and cannot achieve the function of detecting and tracking multiple rats to study their social behavior, more than two rats can be detected and tracked with the framework being proposed. A Rat-YOLO tracking software is developed, which includes a friendly, real-time rat tracking platform and automatically generates labeled datasets. In the framework, nine-point position correction arithmetic is proposed in the fixed scene to correct the wrongly positioned coordinates. The source code was accessed openly from GitHub (Appendix A). A Rat-YOLO model trained on a fixed scene is designed in the project to detect the rat motion track, and the model was tested by a fixed scene which included one rat and another fixed scene which included three rats, the result of which is shown in Supplementary Video S1 recoded from the platform. The framework can output the location of every rat in real time, and after the video is completed, two figures of trajectory and exploration are plotted. Thus, the frame proposed in the study can achieve the goal of multi-rat detecting and tracking in real time and obtaining the activity track map when rats are under a fixed scene. In addition to this, as shown in Figure 9, a software capable of automatically generating labeled datasets is designed to generate labeled datasets in the study.

The traditional software commonly adopted the background subtraction (the marked joint angle of rat, and the result is shown in the Video S2). However, there is a disadvantage in detecting an object from a dynamic background. There is a great advantage in the CNN detection, which ignores the background changes of detected objects. The main advantage is that the YOLO detector [17] is trained on the COCO dataset [35], which includes up to 91 kinds of objects under different backgrounds. Thus, in theory, the software proposed in the paper can achieve multiple types object tracking and we can train the model under other experimental scenes, such as fruit, fish, and people.

## Figures and Tables

**Table 1:** The original image pixel.

$P_{\left(x-2,y-2\right)}$	$P_{\left(x-1,y-2\right)}$	$P_{\left(x,y-2\right)}$	$P_{\left(x+1,y-2\right)}$	$P_{\left(x+2,y-2\right)}$
$P_{\left(x-2,y-1\right)}$	$P_{\left(x-1,y-1\right)}$	$P_{\left(x,y-1\right)}$	$P_{\left(x+1,y-1\right)}$	$P_{\left(x+2,y-1\right)}$
$P_{\left(x-2,y\right)}$	$P_{\left(x-1,y\right)}$	$P_{\left(x,y\right)}$	$P_{\left(x+1,y\right)}$	$P_{\left(x+2,y\right)}$
$P_{\left(x-2,y+1\right)}$	$P_{\left(x-1,y+1\right)}$	$P_{\left(x+1,y+1\right)}$	$P_{\left(x+1,y+1\right)}$	$P_{\left(x+2,y+1\right)}$
$P_{\left(x-2,y+2\right)}$	$P_{\left(x-1,y+2\right)}$	$P_{\left(x+2,y+2\right)}$	$P_{\left(x+1,y+2\right)}$	$P_{\left(x+2,y+2\right)}$

**Table 2:** The 5 × 5 convolution kernel.

$K_{\left(x-2,y-2\right)}$	$K_{\left(x-1,y-2\right)}$	$K_{\left(x,y-2\right)}$	$K_{\left(x+1,y-2\right)}$	$K_{\left(x+2,y-2\right)}$
$K_{\left(x-2,y-1\right)}$	$K_{\left(x-1,y-1\right)}$	$K_{\left(x,y-1\right)}$	$K_{\left(x+1,y-1\right)}$	$K_{\left(x+2,y-1\right)}$
$K_{\left(x-2,y\right)}$	$K_{\left(x-1,y\right)}$	$K_{\left(x,y\right)}$	$K_{\left(x+1,y\right)}$	$K_{\left(x+2,y\right)}$
$K_{\left(x-2,y+1\right)}$	$K_{\left(x-1,y+1\right)}$	$K_{\left(x,y+1\right)}$	$K_{\left(x+1,y+1\right)}$	$K_{\left(x+2,y+1\right)}$
$K_{\left(x-2,y+2\right)}$	$K_{\left(x-1,y+2\right)}$	$K_{\left(x,y+2\right)}$	$K_{\left(x+1,y+2\right)}$	$K_{\left(x+2,y+2\right)}$

**Table 3:** The accuracy of the Rat-YOLO detector.

No Rats Detected	One Rat Detected	Two Rats Detected	Three Rats Detected	More than Three Rats Detected	Total Error Frames after Correction	Detected Rats after Our Framework
25	88	658	2098	23	139	2753
0.864%	3.043%	22.752%	72.545%	0.795%	4.806%	95.194%

## Supplementary Materials

The following are available online at https://www.mdpi.com/1424-8220/20/1/2/s1, the detecting and tracking video (S1: Rats Detecting and Tracking) is available at https://drive.google.com/file/d/1N7mPyDHz5IKXZIWr6Et7TuIRuPqz-vaA/view?usp=sharing. The detecting and tracking video (S2: Marked Joint of Rat Detecting and Tracking) is available at https://drive.google.com/file/d/16Lh7AtgX11Aga-xHcpveILvubs2liMXs/view?usp=sharing.

## Appendix A

The code for the Rat-YOLO is available at https://github.com/lxdfrank/MultiObjectTracking.

The trained weight is available at https://drive.google.com/file/d/15obDyfFsxddkE_BSOVaez-FsunK6Xa0X-/view?usp=sharing.
